# Supplementary material for: R-spondin signalling is essential for the maintenance and differentiation of mouse nephron progenitors
Source: eLife. 2020 May 1;9:e53895. doi: 10.7554/eLife.53895 (PMC7228766; doi:10.7554/eLife.53895)
Supplement: Supplementary file 1. [file elife-53895-supp1.docx]

**Supplementary File 1**

| **Key Resources Table** | | | | |
| --- | --- | --- | --- | --- |
| **Reagent type (species) or resource** | **Designation** | **Source or reference** | **Identifiers** | **Additional information** |
| \| other \| random hexamers \| Thermo Fisher Scientific \| Thermo Fisher Scientific:N8080127 \| \| --- \| --- \| --- \| --- \| | Random hexamers | Thermo Fisher Scientific | \| Cat#: N8080127 \| \| --- \| | RT-PCR reagent |
| other | M-MLV Reverse Transcriptase | Thermo Fisher Scientific | Cat#: 28025013 | RT-PCR reagent |
| other | dNTP set | Thermo Fisher Scientific | Cat#: R0181 | RT-PCR reagent |
| other | Mayer’s Hematoxylin | Sigma Aldrich | Cat#: MHS80 |  |
| other | Normal Donkey Serum | Jackson Immuno Research | Cat#: 017-000-121 | IF (10 % in blocking reagent) |
| other | APEL^TM^2 | STEMCELL Technologies | Cat#: 05270 | Nephron isolation and culture |
| other | PFHM II | ThermoFisher Scientific | Cat#: 12040077 | Nephron isolation and culture |
| other | Vectashield antifade mounting medium with DAPI | Vector Laboratories | Cat#:H1200 |  |
| Commercial assay or kit | Vector® Red Substrate kit, Alkaline Phosphatase | Vector Laboratories | Cat#:SK51000 |  |
| Commercial assay or kit | RNAScope® 2.5AH Assay-red kit | ACD (R&D systems) | Cat#: 322350 | ISH |
| Commercial assay or kit | RNAeasy mini kit | Qiagen | Cat#: 74104 | RNA isolation |
| Commercial assay or kit | RNAeasy micro kit | Qiagen | Cat#: 74004 | RNA isolation |
| Commercial assay or kit | *In situ* cell death detection kit, TMR red | Roche | Cat#: 12156792910 |  |
| Chemical compound drug | Corn Oil | Sigma Aldrich | Cat#: C8267 |  |
| Chemical compound drug | Paraformaldehyde (16% solution) | Electron Microscopy Science | Cat#: 15710 | Tissue fixation (dil. 1:4 dil) |
| Chemical compound drug | Tween 20 | Sigma Aldrich | Cat#: P9416 | IF (0.01%) |
| Chemical compound drug | Tamoxifen | Sigma Aldrich | Cat#: T5648 | For IP (200 mg/kg of body weight) |
| Chemical compound drug | Heparin | Sigma Aldrich | Cat#: H3149 | Nephron isolation and culture (1 μg/ml) |
| Chemical compound drug | BMP purple | Sigma Aldrich | Cat#: 11442074001 |  |
| Chemical compound drug | Glycerol | Sigma Aldrich | Cat#: G5516 |  |
| Chemical compound drug | Eosin Y | Sigma Aldrich | Cat#: E4009 |  |
| Chemical compound drug | Light Cycler® 480 SYBR Green I Master Kit | Roche Diagnostics (Sigma Aldrich) | Cat#: 04707516001 |  |
| peptide, recombinant protein | mRSPO3 | R&D Systems | Cat#: 4120RS | MET test (200ng/ml) |
| peptide, recombinant protein | WNT3a | R&D Systems | Cat#: 5036-WN | MET test (50ng/ml) |
| Chemical compound drug | CHIR 99021 | TOCRYS | Cat#: 4423 | MET test (3 μg/ml) |
| peptide, recombinant protein | rh-FGF9 | R&D Systems | Cat#: 273-F9 | Nephron isolation and culture (200ng/ml) |
| strain, strain background (*Mus musculus*) | Lgr4/5/6 triple mutant | Szenker-Ravi E, et al.  PMID: 29977062 |  | Use of both male and female |
| strain, strain background (*Mus musculus*) | Ctnnbflox | Brault V, et al.  PMID: 11262227 | RRID:MGI:2674117 | Use of both male and female |
| strain, strain background (*Mus musculus*) | CAGG:Cre-ERTM | Hayashi S. et al.  PMID: 11944939 |  | Use of both male and female |
| strain, strain background (*Mus musculus*) | Wt1:CreERT2 | Zhou B, et al.  PMID: 18568026 | RRID:MGI:3802698 | Use of both male and female |
| strain, strain background (*Mus musculus*) | Six2:Cre | Kobayashi A. et al.  PMID: 18682239 | RRID:MGI:3848499 | Use of both male and female |
| strain, strain background (*Mus musculus*) | Foxd1:Cre | Kobayashi A. et al.  PMID: 25358792 | RRID:IMSR_JAX:012463 | Use of both male and female |
| strain, strain background (*Mus musculus*) | Rspo1- | Chassot AA, et al.  PMID: 18250098 | RRID: MGI:3795283 | Use of both male and female |
| strain, strain background (*Mus musculus*) | Rspo3flox | Rocha AS, et al.  PMID: 26655896 | RRDI: MGI: 3696850 | Use of both male and female |
| strain, strain background (*Mus musculus*) | Lgr4- | Da Silva F, et al.  PMID: 28834739 |  | Use of both male and female |
| biological sample (*Mus musculus*) | Mouse embryos | Institute of Medical Biology, A*STAR Singapore | Lgr4/5/6 triple knockout and control kidneys | Freshly isolated from *Mus musculus* were fixed, dehydrated and embedded in paraffin blocks |
| biological sample (*Mus musculus*) | Mouse embryos or mouse embryonic tissus | Institut de Biologie Valrose, Nice, France | All other mouse samples analysed in this paper | Freshly isolated from *Mus musculus*, were either fixed, dehydrated and embedded in paraffin blocks, or directed snap frozen in liquid nitrogen for further RNA extraction. |
| antibody | ALDH1A2 (Rabbit polyclonal) | Sigma-Aldrich | Cat# HPA010022,  RRID:AB_1844723 | IF dil (1:300) |
| antibody | anti-BrdU  (clone 3D4)  (Mouse monoclonal) | BD Bioscience | Cat# 555627,  RRID:AB_395993 | IF dil (1:250) |
| antibody | anti-CDH1  anti-E CADHERIN  (clone36/E)  (Mouse monoclonal) | BD Bioscience | Cat#: 610181,  RRID:AB_2076677 | IF dil (1:500) |
| antibody | Anti-FOXD1  (Goat polyclonal) | Santa Cruz | Cat#: sc-47585,  RRID:AB_2105295 | IF dil (1:100) |
| antibody | Anti-JAGGED1  (Goat polyclonal) | Santa Cruz | Cat#: sc-6011,  RRID:AB_649689 | IF dil (1:200) |
| antibody | Anti-LEF1  (clone EPR2029Y)  (Rabbit monoclonal) | Abcam | Cat#: ab137872 | IF dil (1/250) |
| antibody | Anti-MEIS 1  (Goat polyclonal) | Sigma-Aldrich | Cat#: sab2500627,  RRID:AB_10605014 | IF dil (1:250) |
| antibody | Anti-NPHS1  Anti-NEPHRIN  (Goat polyclonal) | R&D Systems | Cat#: AF3159,  RRID:AB_2155023 | IF dil (1:250) |
| antibody | Anti-P-SMAD1/5  (Rabbit polyclonal) | Cell Signalling | Cat#:9516 | IF dil (1:200) |
| antibody | Anti-SIX2 (Rabbit polyclonal) | Proteintech | Cat#: 11562-1-AP,  RRID:AB_2189084 | IF dil (1:300) |
| antibody | Anti-WT1  ((Clone 6F-H2)  (Mouse monoclonal) | DAKO | Cat#: M3561 | IF dil (1:300) |
| antibody | Anti-Digoxigenin-AP, Fab fragments | Roche | Cat#: 11093274910,  RRID:AB_514497 | ISH dil (1:5000) |
| recombinant DNA reagent | pCRII Topo vector | Invitrogen | Cat#: K465040 |  |
| sequence-based reagent | Mm-Axin2 | ACD  RNAScope® probe | Cat#: 400331 | ISH |
| sequence-based reagent | *Mm-*Lgr4 | ACD  RNAScope® probe | Cat#: 318321 | ISH |
| sequence-based reagent | Mm-Lgr5 | ACD  RNAScope® probe | Cat#: 312171 | ISH |
| sequence-based reagent | Mm-Lgr6 | ACD  RNAScope® probe | Cat#: 404961 | ISH |
| sequence-based reagent | Mm-Rspo1-01 | ACD  RNAScope® probe | Cat#: 479591 | ISH |
| sequence-based reagent | Mm-Rspo2 | ACD  RNAScope® probe | Cat#: 402001 | ISH |
| sequence-based reagent | Mm-Rspo3- | ACD  RNAScope® probe | Cat#: 402011 | ISH |
| software, algorithm | Halo | Indica labs |  |  |
| software, algorithm | Adobe Photoshop | Adobe systems | RRID:SCR_014199 |  |
| software, algorithm | Fiji | https://fiji.sc | RRID:SCR_002285 |  |
| software, algorithm | GraphPad Prism 5.0 | https://www.graphpad.com | RRID:SCR_002798 |  |
